# Assessing Extracellular Vesicle Turnover In Vivo Using Highly Sensitive Phosphatidylserine‐Binding Reagents

**DOI:** 10.1002/advs.202507624

**Published:** 2025-08-16

**Authors:** Lavinia Flaskamp, Monica Prechtl, Annkathrin Scheck, Wenbo Hu, Christine Ried, Georg Kislinger, Mikael Simons, Anne B. Krug, Jan Kranich, Thomas Brocker

**Affiliations:** ^1^ Institute for Immunology Faculty of Medicine BMC, LMU Munich Großhaderner Strasse 9 82152 Planegg Germany; ^2^ German Center for Neurodegenerative Diseases (DZNE) Munich Germany

**Keywords:** EV‐turnover, extracellular Vesicles (EVs), lactadherin, MFG‐E8, phosphatidylserine (PS)

## Abstract

Extracellular vesicles (EVs) are emerging as crucial players in cell communication and hold great promise as biomarkers and therapeutic tools. However, their diversity makes it challenging to detect, classify, and utilize them effectively, which limits their clinical applicability. A key challenge is the lack of reliable markers to identify EVs consistently. In this study, a novel high‐affinity phosphatidylserine (PS)‐binding reagent is introduced for EV analysis. PS is known as a marker of apoptotic cells and activated platelets, but its presence on EVs is debated due to variations in lipid composition. By comparing multiple PS‐binding reagents, including MFG‐E8 derivatives and Annexin V, it is demonstrated that ≈90% of EVs in human and mouse blood carry PS. Using the optimized reagent, the first in vivo insights into EV turnover are provided, showing that PS+ EVs in mouse blood are rapidly cleared (≈50% within 30 min) but persist on immune cells in the spleen. This discovery increases the potential of EVs as disease biomarkers and therapeutic targets by improving EV detection and isolation as well as opening the door for standardized quantification and diagnostic monitoring.

## Graphical abstract

Extracellular vesicles (EVs) are promising biomarkers, but their heterogeneity limits clinical use. A novel high‐affinity phosphatidylserine (PS)‐binding reagent is introduced that reveals ≈90% of circulating EVs are PS⁺. This enables in vivo tracking, showing rapid EV clearance and retention in spleen immune cells—advancing EV quantification, detection, and clinical application.

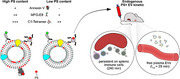

## 1 Introduction

Extracellular vesicles (EVs) have emerged as critical mediators of intercellular communication and hold great promise as biomarkers and therapeutic agents.^[^ ^1^, ^2^ ^]^ Over the past two decades, EV research has expanded rapidly, revealing their roles in physiological and pathological processes, as well as their potential as cargo‐delivery systems in therapy.^[^ ^3^ ^]^ However, despite this growing interest, the field faces major challenges in EV characterization and standardization.^[^ ^4^ ^]^ A universal EV marker has yet to be identified, making it difficult to reliably detect and classify these particles across different biological contexts.

Phosphatidylserine (PS), a negatively charged phospholipid, is typically confined to the inner leaflet of cellular membranes.^[^ ^5^ ^]^ However, during apoptosis, PS is externalized due to the inactivation of flippases and activation of scramblases, contributing to membrane disintegration and EV release.^[^ ^6^, ^7^, ^8^ ^]^ While PS has been implicated as a marker for apoptotic bodies and certain EV subsets, its distribution across different EV populations remains debated.^[^ ^9^, ^10^, ^11^, ^12^, ^13^ ^]^ Some studies report that only tumor‐derived EVs are PS^+ [^ ^14^, ^15^, ^16^, ^17^, ^18^ ^]^ while others suggest that PS is present on the majority of EVs, albeit at varying levels depending on their source.^[^ ^19^ ^]^ The lack of a highly sensitive, standardized PS‐specific reagent has further compounded these discrepancies.

To address this, we systematically compared multiple PS‐binding reagents‐including a novel recombinant MFG‐E8‐eGFP, SA‐tetramerized MFG‐E8 C1 domain (C1‐tetramer), and Annexin V‐to evaluate their ability to detect free blood EVs. Using imaging flow cytometry (IFC), bead‐assisted flow cytometry, and super‐resolution microscopy, we found that over 90% of circulating and cell‐bound EVs in both murine and human blood are PS+. Notably, we observed significant differences in the affinities of PS‐binding proteins, which likely account for inconsistencies in previous reports.

We further leveraged our most sensitive reagent, C1‐tetramer, for in vivo EV tracking, providing the first direct insights into endogenous EV turnover. Our data reveal that murine PS+ plasma EVs are rapidly cleared from circulation (t_1/2_ ≈30 min) but persist on immune cells in the spleen, demonstrating a dynamic EV lifecycle. These findings establish PS as a broad EV marker and offer a standardized approach for EV detection, quantification, and functional analysis. Given the growing interest in EVs across biomedical fields, our work provides a critical tool for advancing both fundamental research and translational applications.

## 2 Results

### 2.1 Comparison of PS‐Binding of Different PS‐Specific Reagents

**Figure 1:**
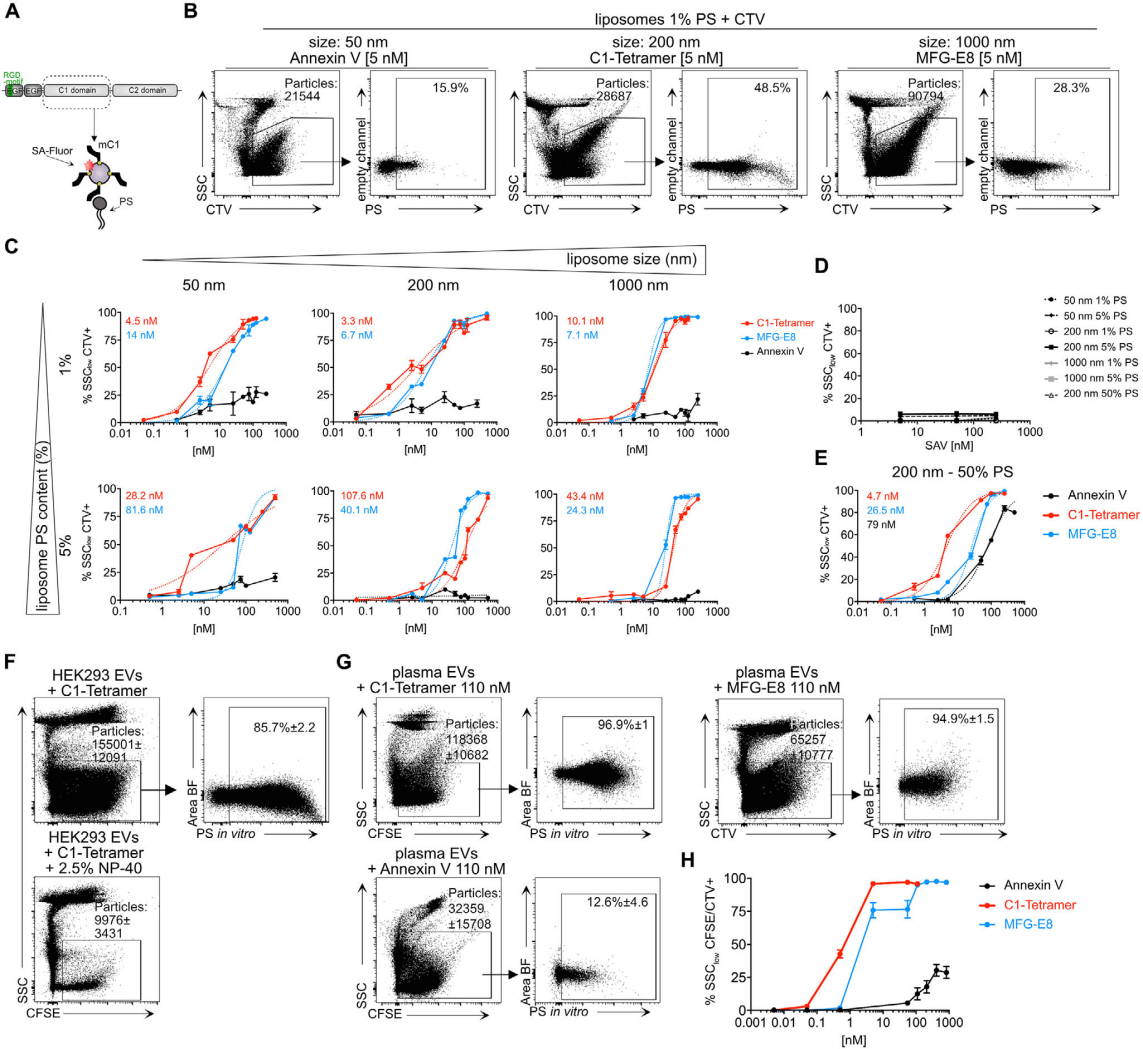
MFG‐E8‐based PS‐binding reagents efficiently stain small liposomes with low PS content and murine plasma EVs. A) Schematic representation of full‐length MFG‐E8 domains and the C1‐tetramer produced by tetramerization of the monomeric biotinylated C1 domain (mC1) with fluorescently conjugated streptavidin (SA‐Fluor). B) Imagestream analysis of liposomes (Encapsula), shown is the exemplary gating strategy for 50, 200, and 1000 nm‐sized liposomes, using the membrane dye cell‐trace violet (CTV) and excluding high scatter events. C) Liposomes with a specific size and lipid ratio (DOPC:DOPS 99:1 or 95:5) were used to find out how well Annexin V‐AF488 (black), full‐length MFG‐E8‐eGFP (blue), or the C1‐tetramer‐AF488 (red) bound to them. All samples were measured as technical duplicates or triplicates. D) binding specificity of the C1‐tetramer was assessed by incubating liposomes with increasing concentrations of SA‐AF488. E) liposomes (200 nm) with high PS content (DOPC:DOPS, 1:1) labeled with distinct concentrations of Annexin V‐AF488 (black), full‐length MFG‐E8‐eGFP (blue), or the C1‐tetramer‐AF488 (red). Dissociation constants (kD) were calculated by fitting non‐linear regression curves with the Hill slope equation. F) Cell culture‐derived EVs were isolated from the supernatant of serum‐free adapted HEK293 cells (n = 4) and analyzed by IFC for PS positivity, exemplary gating on EVs is shown using the membrane dye carboxyfluorescein succinimidyl ester (CFSE) and excluding high scatter events. G) Sensitivity of different PS‐binding proteins for PS+ murine plasma EVs was examined by IFC; exemplary gating on EVs is shown using CFSE or CTV and excluding high scatter events. H) PS‐labeling efficiency of membrane particles at distinct concentrations of Annexin V (AF647), C1‐tetramer (AF647), or MFG‐E8‐eGFP (n = 3).

We have developed recombinant PS‐binding proteins, including i) full‐length MFG‐E8, ii) MFG‐E8 C1C2, which lacks the epidermal‐growth factor (EGF) like domain and the proline‐threonine (PT)‐rich domain, and iii) streptavidin (SA)‐multimerized MFG‐E8 C1 domains (C1‐tetramer; **Figure** **1**A), to differentiate between PS^+^ live cells associated with PS^+^ extracellular vesicles (EVs) and authentic PS^+^ apoptotic cells.^[^ ^20^, ^21^, ^22^ ^]^ While these MFG‐E8 derivatives proved useful to detect cell‐bound EVs, it was unclear which frequencies of free (unbound) EVs were PS^+^ and thus detectable. Historically, PS‐binding proteins like Annexin V and purified bovine MFG‐E8 have been applied to label EV preparations, though these often produced heterogeneous results. Considering the variability in PS content ^[^ ^19^ ^]^ and EV sizes, we aimed to assess whether different reagents are equally effective for detecting PS^+^ EVs.

To this end, we first conducted experiments using liposomes of various sizes (50, 200, and 1000 nm) with defined PS contents of 1% and 5% (Figure 1B,C). We then analyzed the labeling efficiency of these liposomes by fluorescently conjugated PS‐binding proteins using imaging flow cytometry (IFC), following an initial calibration (Figure S1A, Supporting Information), as previously described.^[^ ^23^ ^]^ For all single particle flow cytometry experiments, we adhered to MiFlowCyt recommendations for controls, with detailed tables (Tables S1 and S2, Supporting Information) and figures available in the supplement.^[^ ^24^ ^]^

For liposome labeling, we used the membrane dye CellTrace Violet (CTV) to determine the percentage of PS positivity across all detected particles (Figure 1B; Figure S1B–F, Supporting Information). This analysis revealed notable differences between MFG‐E8 derivatives and Annexin V; the latter failed to label significant portions of any liposome populations tested (Figure 1C). Furthermore, Annexin V's labeling efficiency plateaued at 25% for small liposome types, even at higher concentrations. In contrast, both full‐length MFG‐E8 and the C1‐tetramer achieved nearly 100% labeling efficiency, regardless of liposome size or PS content (Figure 1C).

Notably, the C1‐tetramer demonstrated higher sensitivity (kD) than full‐length MFG‐E8 for the smallest (50 nm, 1% and 5% PS) and medium‐sized (200 nm, 1% PS) liposomes (Figure 1C). This aligns with prior findings suggesting that curvature and PS content modulate the binding efficiency of full‐length MFG‐E8.^[^ ^25^, ^26^ ^]^ Importantly, the C1‐multimer, comprising the minimal PS‐binding C1 domain of MFG‐E8, extends applicability to even the smallest vesicles with low PS content (Figure 1C), while fluorescently labeled SA alone, the backbone of the multimer, did not bind (Figure 1D). However, Annexin V labeled close to 90% of the high PS frequency 200 nm liposomes (50% PS content) in our analysis (Figure 1E), albeit still with the lowest affinity compared to MFG‐E8 and the C1‐tetramer.

Next, we extended our analysis to HEK293 cell culture supernatant‐derived EVs (Figure S1G,H, Supporting Information) by IFC^[^ ^24^ ^]^ to serve as reference EV material. EVs were defined as membrane dye‐positive (CFSE or CTV) and side scatter (SSC) low (Figure 1F; Figures S1I–L and S2D, Supporting Information), with detergent sensitivity further confirming their membranous nature. This analysis revealed that the great majority of HEK293 EVs were PS+ (85.7 ± 2.2%). Using CD9 as an additional EV marker, we found that 90.9% of the tetraspanin‐positive HEK293 EVs are also PS+ (Figure S1J, Supporting Information).

We then tested these PS‐binding proteins on EVs purified from murine and human blood, where PS levels are typically very low,^[^ ^19^ ^]^ making reagent sensitivity particularly crucial. Plasma EVs were isolated from mouse blood through sequential centrifugation and size‐exclusion chromatography (SEC), with EV‐containing fractions identified by nanoparticle tracking analysis (NTA), protein quantification (bicinchoninic acid assay), and Western blotting (Figure S2A,B, Supporting Information). The isolated plasma EVs had a mean size of 152 ± 10.2 nm, which remained unchanged when EVs were labeled with the C1‐tetramer (146 ± 5.6 nm; Figure S2C, Supporting Information).

EVs were subsequently stained with increasing concentrations of Annexin V, recombinant full‐length murine MFG‐E8, or the C1‐tetramer to assess the frequency of PS^+^ EVs (Figure 1H; Figures S2E–G and S3A–C, Supporting Information). Our findings showed significant differences in PS^+^ EV detection across reagents, consistent with prior liposome results. At the highest concentration, Annexin V labeled only ≈28% of EVs, while both the C1‐tetramer and MFG‐E8 detected nearly all EVs (≈95–97%, Figure 1H). Notably, the C1‐tetramer achieved maximum detection levels at concentrations 166 times lower than those required for Annexin V. In contrast, MFG‐E8 required concentrations 22 times higher than the C1‐tetramer to reach similar detection levels, highlighting the superior affinity of the latter (Figure 1H).

Previous studies, including our own, have demonstrated that MFG‐E8 derivatives and Annexin V exhibit similar detection capacities for PS^+^ dead cells^[^ ^20^, ^22^, ^27^ ^]^ (Figure S2H, Supporting Information). Indeed, when we used dead cells, the C1‐tetramer stained a greater proportion of cells (81.3 ± 2.2%) compared to Annexin V (66.9 ± 2.3%) at the highest concentration (Figure S4A–C, Supporting Information), while on PS^hi^ necrotic (LD^+^) cells, differences among Annexin V, MFG‐E8, and the C1‐tetramer were small (Figure S4B, Supporting Information). This suggests that reagent efficacy depends on PS‐content of the target structure.

Also, the median fluorescence intensity (MFI) for PS on spleen cells (Figure S4C, Supporting Information) revealed differences in PS‐binding protein sensitivity when focusing on live (LD^−^) splenocytes. Here, the C1‐tetramer showed the highest sensitivity as LD^–^ splenocytes encompass apoptotic and EV‐decorated cells, which generally exhibit less PS exposure than necrotic cells (Figure S4C, Supporting Information).^[^ ^20^ ^]^

Together, these findings highlight the confounding effects of using PS‐staining reagents with varying sensitivities, a factor especially pronounced in contexts with low PS expression, such as on blood EVs. For Annexin V, only a subset of EVs was detected, likely representing EVs with high PS exposure, while MFG‐E8 appears more effective in binding EVs with both high and low PS frequencies (Figure 1C–H). Interestingly, PS exposure emerges as a dominant feature of plasma EVs when using the C1‐tetramer and, despite its lower sensitivity, also with MFG‐E8.

### 2.2 PS Exposure is a Common Characteristic Across all EV Populations in Mice

Given the heterogeneous nature of blood‐derived EVs, we aimed to further characterize PS^+^ EVs and differentiate them from potential contaminants.

**Figure 2:**
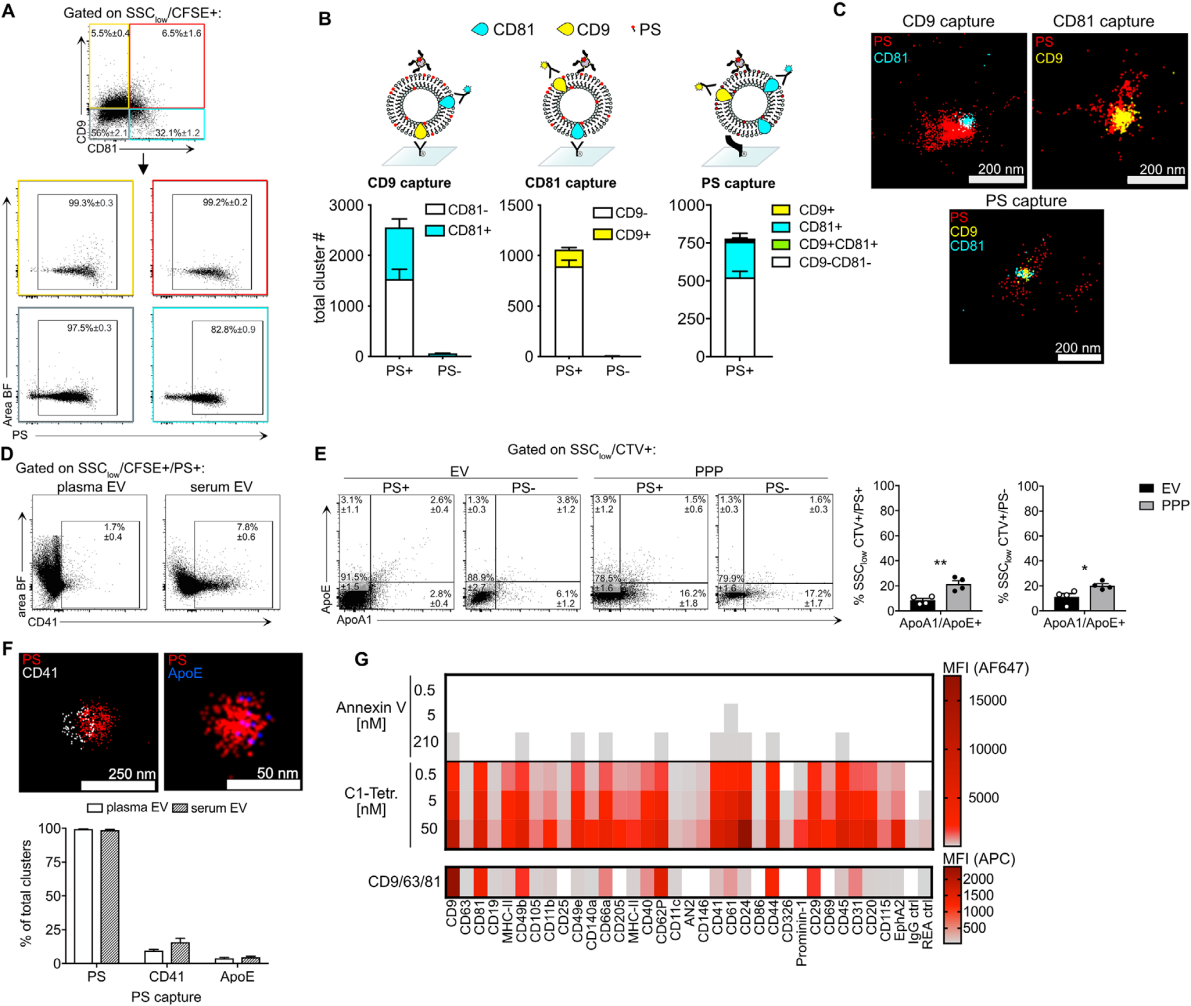
C1 tetramers stain various EV populations. A) IFC anaylsis for tetraspanins CD9 and CD81 on murine plasma EVs (n = 3), PS positivity of distinct EV subsets was examined. B) Schematic representation of CD9‐, CD81‐, and PS‐based capturing for superresolution microscopy of EVs (top row). EVs were stained for CD81 and/or CD9 and PS and then captured onto SA‐coated glass slides with anti‐CD9‐biotinylated, anti‐CD81‐biotinylated antibodies or biotinylated mC1, respectively. Plasma EVs stained for PS and CD9 or CD81 were examined by superresolution microscopy and cluster analysis. Bar graphs show counts of PS+ or PS‐ clusters (PS+, PS‐), CD81 (cyan), CD9 (yellow), or CD9/CD81 double positive (green). Data (n = 5) are shown as mean ± SEM. Exemplary dSTORM micrographs are shown in (C). D) IFC analysis of SSC_low_/CFSE+/PS+ murine plasma or serum EVs (n = 3) for CD41 as a platelet marker. E) IFC analysis of murine plasma EVs purified via SEC ('EV') versus platelet‐poor‐plasma (PPP), apolipoprotein markers (ApoA1, ApoE) were used to test for lipoprotein contamination in the respective samples (n = 4). Total ApoA1/E1+ detected particles are depicted in bar graphs for PS+ and PS‐ events. Normality of the data was assessed by Shapiro‐Wilk normality test, and statistical significance was tested using unpaired *t*‐test (*p*< 0.05, ^*^. *p*< 0.005,^**^). F) Superresolution microscopy and cluster analysis using PS capture. Representative micrographs are shown to the left. Bar graphs (right) show percentages of PS+, CD41+, and ApoE+ clusters in plasma and serum. Error bars represent ±SEM. G) The upper panel shows surface exposure of PS analyzed by bead‐assisted flow cytometry, using the C1‐Tetramer‐AF647 or Annexin V‐AF647 for PS staining. Distinct EV subpopulations were captured by bead‐coupled antibodies targeting the indicated proteins (columns). Color coding indicates MFI for AF647 of the respective bead population, MFI values were background corrected by a 'no EV' blank control, and for MFI values obtained for C1‐Tetramer‐AF647 or Annexin V in PBS (no EVs) for the C1‐Tetramer and Annexin V staining, respectively. Displayed are means of biological replicates (n = 3). Lower panel shows surface exposure of CD9/CD63/CD81 analyzed by bead‐assisted flow cytometry, using a detection antibody mix (Miltenyi). Distinct EV subpopulations were captured by bead‐coupled antibodies targeting the indicated proteins (columns). Color coding indicates MFI for APC of the respective bead population, MFI values were background corrected by a 'no EV' blank control.

Tetraspanins such as CD9, CD63, and CD81, which are enriched on exosomes and other EVs, are often considered universal EV markers.^[^ ^28^ ^]^ Therefore, we examined PS positivity among tetraspanin^+^ EV populations using CD9/CD81 antibodies and IFC (Figure 2A). When gating on tetraspanin^+^ EV subsets, the proportion of PS^+^ events ranged from 82.8% to 99.2%, representing most of the distinct EV populations. To confirm these results, we employed direct stochastic optical reconstruction microscopy (dSTORM), achieving a resolution of ≈20 nm, to evaluate PS positivity in specific EV populations.

We immobilized plasma EVs on glass slides, and schematic representations of the different capture and detection strategies are shown in Figure 2B (top panel). We used either biotinylated anti‐CD9 to capture CD81 and PS‐labeled EVs (Figure 2B, left) or anti‐CD81 to capture CD9 and PS‐labeled EVs (Figure 2B, right). Cluster analysis was conducted with isotype control antibodies and buffer as negative controls (Figure S5D, Supporting Information).

Capturing EVs with any of these antibodies confirmed the findings from IFC (Figures 1G, **2**A), as nearly all CD9‐ and CD81‐captured EVs were PS+ (Figure 2B, lower bar graphs). Only very few PS‐negative EVs could be identified (Figure 2B, PS^‐^).

We also analyzed EVs carrying the platelet marker CD41 to determine whether blood preparation methods (serum vs. plasma) could promote artificial release of platelet‐derived EVs. We observed an increased frequency of CD41^+^ PS+ events in serum EV preparations (Figure 2D; Figure S5E–H, Supporting Information) following coagulation, whereas in plasma EVs, only a small fraction of PS+ events were CD41^+^ (1.7 ± 0.4%). As lipodomic analyses showed that PS can be enriched in small dense HDL,^[^ ^29^ ^]^ we assessed the amount of PS+ lipoproteins in our EV preparations. For this, we used IFC and super‐resolution microscopy to quantify ApoA1 and ApoE, markers found on murine HDL, LDL, VLDL, and chylomicrons.^[^ ^30^ ^]^ We compared SEC purified plasma EVs with platelet‐poor plasma (PPP) and found that levels of both markers were significantly reduced after SEC (Figure 2E; Figure S6A–E, Supporting Information). In total, we found less than 10% ApoA1/E+ particles (9 ± 1.5%) among detected PS+ events in our SEC purified plasma EVs. Super‐resolution microscopy supported these observations, showing ApoE present in only 4 ± 0.6% and 5 ± 0.6% of clusters in plasma and serum EV preparations, respectively (Figure 2F; Figure S6I, Supporting Information).

To further investigate PS labeling differences, we compared Annexin V and C1‐tetramer using a commercially available multiplex bead assay that analyzes 37 distinct EV populations based on surface protein capture. Exposed PS on captured plasma EVs was detected with Annexin V‐AF647 or C1‐tetramer‐AF647 (Figure 2G; Figure S6F–H, Supporting Information). Staining with C1‐tetramer revealed PS signal above background for all EV populations except CD326^+^ EVs, regardless of concentration. This supports the notion that PS exposure is a predominant feature of diverse EV types. However, signal intensities among populations may also reflect differences in vesicle capture rather than PS content alone. In contrast, PS staining with Annexin V required a concentration four times higher than C1‐tetramer, and even then, only one‐third of EV bead populations showed detectable PS. Notably, the Annexin V‐positive populations aligned with those showing the highest C1‐tetramer signal intensities, such as CD9 (Figure 2G). Moreover, when comparing the presence of PS with the presence of tetraspanins on the different EV populations, we found that PS was detectable on many more EV populations than tetraspanins (Figure 2G).

Overall, our results demonstrate that PS exposure is a defining feature of blood EV populations. Importantly, in our isolated plasma EV preparations, only a small fraction (4–9%) of PS^+^ particles could be attributed to common contaminants like lipoproteins or platelets. Additionally, this study highlights the labeling bias introduced by varying PS‐binding protein affinities, as evidenced by differing efficiencies of Annexin V and C1‐tetramer in EV population multiplex assays.

### 2.3 The Majority of Human Plasma EVs Similarly Exhibit PS Exposure

We next determined whether human plasma EVs exhibit similar PS‐positivity as observed in mice. EV isolation and validation followed the protocol established for murine samples (Figure S7A–C, Supporting Information).

**Figure 3:**
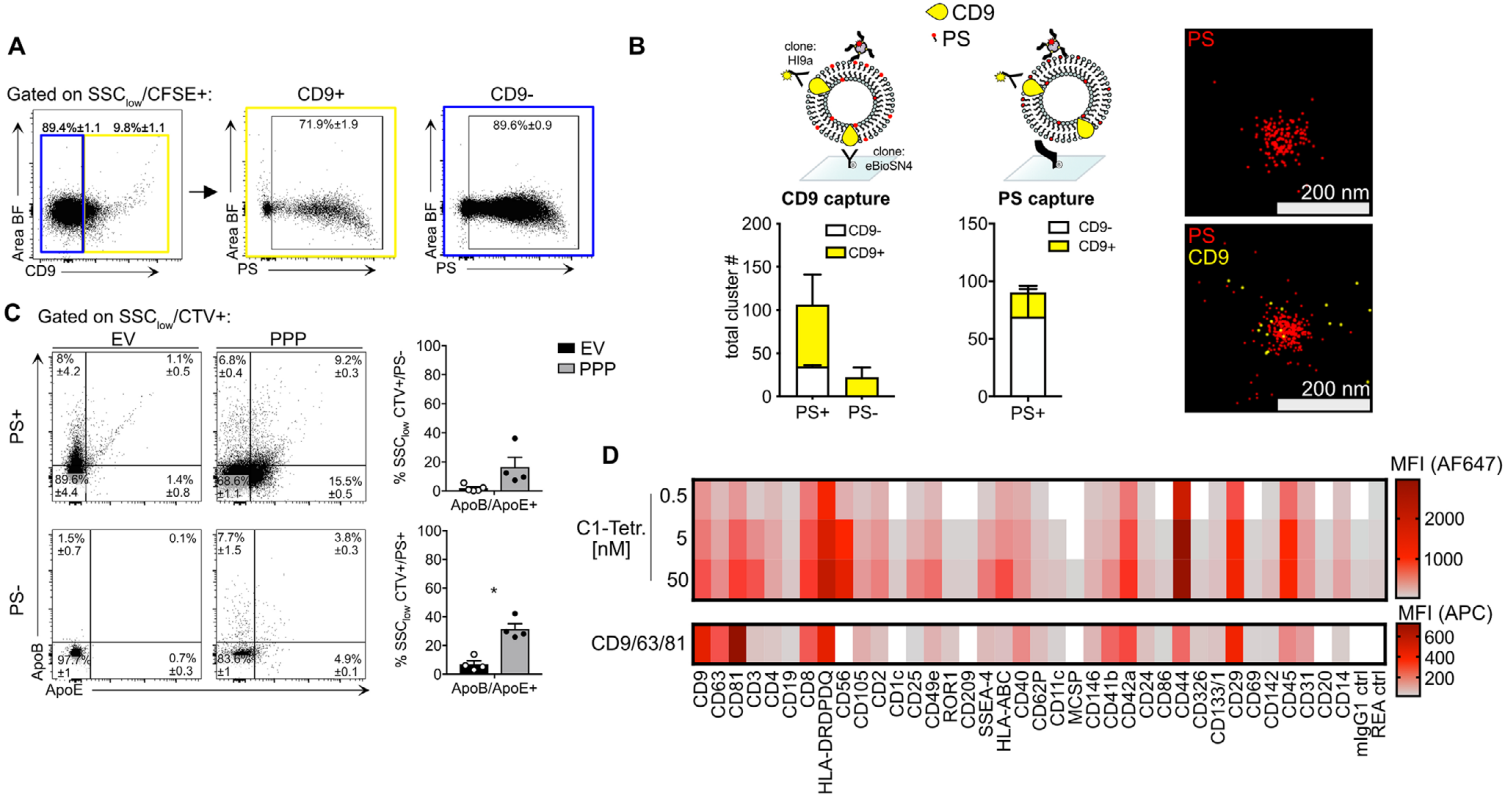
Almost all human plasma EVs are PS+. A) Human plasma EVs (n = 5) were isolated and analyzed by IFC for PS positivity of CD9+ and CD9‐ EVs. Gating on SSC_low_CFSE+CD9+ (yellow box) and CD9‐ EVs (blue box) followed by analysis of PS positivity. Percentages (±SEM) of PS+ EVs are depicted within the gate. B) Schematic representation of CD9‐capturing (biotinylated anti‐CD9 antibody, clone: eBioSN4) or PS‐based capturing (biotinylated mC1) for superresolution microscopy of EVs (upper panel). Human plasma EVs were captured onto SA‐coated glass slides (n = 3) and stained for the tetraspanin CD9 (anti‐CD9 clone: HI9a) and PS (mC1‐tetramer). Bar graphs show counts of PS‐positive clusters (left) and PS‐negative clusters (right). Data are shown as mean±SEM (n = 3). C) IFC analysis of human plasma EVs purified via SEC ('EV') versus PPP, apolipoprotein markers (ApoB, ApoE) were used to test for lipoprotein contamination in the respective samples (n = 4). Total ApoB/E1+ detected particles are depicted in bar graphs for PS+ and PS‐ events. Normality of the data was assessed by Shapiro‐Wilk normality test, and statistical significance was tested using the Mann‐Whitney U or Welch's *t*‐test, respectively (*p*< 0.05, ^*^). D) Surface exposure of PS analyzed by bead‐assisted flow cytometry, using the C1‐Tetramer‐AF647 for PS staining. Distinct EV subpopulations were captured by bead‐coupled antibodies targeting the indicated proteins (columns). Fluorescently conjugated streptavidin (SA) served as a negative control to test the specificity of C1‐Tetramer labeling. Color coding indicates MFI for AF647 of the respective bead population, MFI values were background corrected by a 'no EV' blank control and for MFI values obtained for C1‐Tetramer‐AF647 in PBS (no EVs). Displayed are means of biological replicates (n = 3) for the C1‐Tetramer staining. Lower row, surface exposure of CD9/CD63/CD81 (row) analyzed by bead‐assisted flow cytometry, using a detection antibody mix (Miltenyi). Distinct EV subpopulations are shown which were captured by bead‐coupled antibodies targeting the indicated proteins (columns). Color coding indicates MFI for APC of the respective bead population MFI values were background corrected by a 'no EV' blank control. Displayed are means of biological duplicates (n = 2).

Consistent with murine data, the majority of SSC_low_CTV^+^ events in human plasma EVs were PS^+^ (84.7±1.6%). Upon comparing CD9^+^ and CD9^–^ EV populations by IFC, we observed slightly lower PS levels in CD9^+^ EVs (71.9±1.9%) than in CD9^–^ EVs (89.6 ± 0.9%; **Figure** **3**A; Figure S7D–F, Supporting Information). This trend was further confirmed using super‐resolution microscopy, where 84±4.2% of the CD9‐captured EV clusters were PS‐positive (Figure 3B; Figure S7G, Supporting Information). Similarly to murine samples, we also analyzed human plasma EVs versus PPP for potential lipoprotein contaminations using ApoB and ApoE as markers (Figure 3C; Figure S7H–K, Supporting Information). Among the SEC purified plasma EVs from fasting donors, ApoB/E+ events were almost entirely absent in the PS‐ subset (0.9 ± 0.4%) and significantly reduced among PS+ particles (6.9 ± 2.3%) compared to PPP (31.4 ± 1.1%). While we did not completely remove lipoproteins from our EV preparations, we could demonstrate that they are significantly reduced and make up only a minor fraction of the PS+ particles we detect here.

A multiplex analysis of human plasma EVs detected PS signals above background in 36 out of 37 EV bead populations, except for EVs captured using anti‐melanoma‐associated chondroitin sulfate proteoglycan (MCSP; Figure 3D; Figure S8, Supporting Information). This multiplex assay included general EV markers, such as the tetraspanins CD9, CD63, and CD81, as well as markers indicative of cellular origin (e.g., CD3, CD4, CD8, CD11c, CD45) and cell activation (e.g., CD44). In line with murine plasma EVs, PS was also found to be more universally distributed on human plasma EV populations than CD9/CD63/CD81 (TSPN detection mix, Miltenyi) (Figure 3D). These findings highlight both the heterogeneity and the prevalence of PS^+^ EVs in human plasma samples, mirroring observations in murine models.

### 2.4 Kinetics of Endogenous PS^+^ EVs Determined by In Vivo Labeling

**Figure 4:**
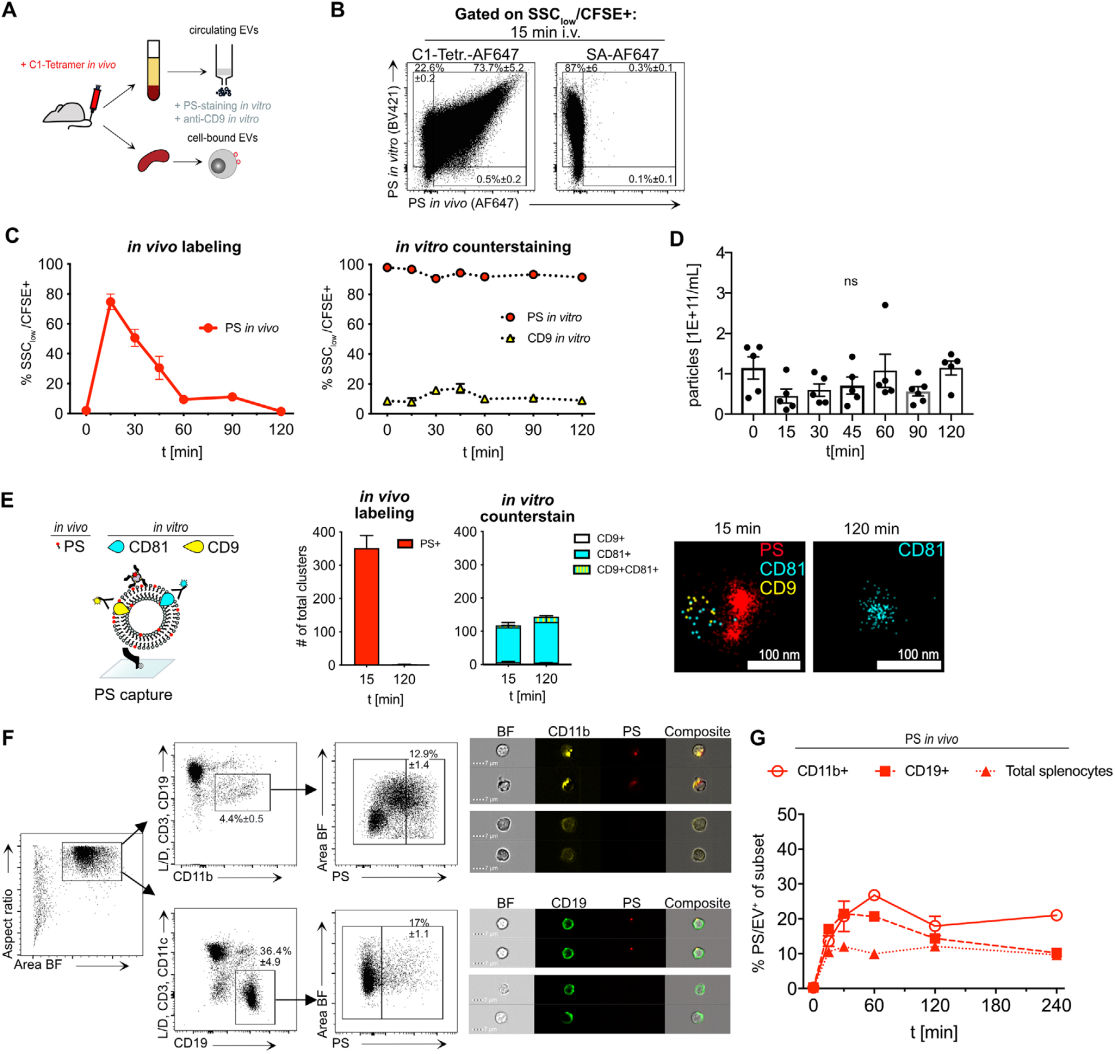
In vivo measurement of EV turnover. A) Schematic illustration of in vivo PS‐labeling. EVs were first labeled in vivo by injection of 50 µg C1‐tetramer‐AF647 i.v. into the animal. After isolation at different time points, plasma EVs were identified by CFSE‐labeling (Figure S9A, Supporting Information). SSC_low_/CFSE^+^ EVs (for gating and EV‐controls, see Figure S9A–C, Supporting Information) were further stained in vitro with anti‐CD9 and C1‐tetramer‐BV421 for multicolor IFC analysis. Furthermore, splenocytes were collected at different time points and analyzed by IFC. B) Dot plots show in vivo vs in vitro PS‐labeled plasma EVs (SSC_low_/CFSE^+^) after 15 min post‐i.v. injection of the C1‐tetramer‐AF647 (left) or SA‐AF647 as a negative control (right). C) Kinetic of in vivo PS‐labeled EVs analyzed by IFC at 15, 30, 45, 60, and 120 min after C1‐tetramer injection. Graphs show percentage of in vivo (left) and in vitro (right) counterstained SSC_low_CFSE+ EVs at different time points (Data are shown as mean ± SEM, n = 4) D) Nanoparticle tracking analysis quantification of particle concentrations in plasma samples at different timepoints post‐injection of the C1‐tetramer. E) Schematic representation (left) of PS‐based capturing for superresolution microscopy of EVs. Briefly, stained EVs were captured onto SA‐coated glass slides using biotinylated mC1 and examined by superresolution microscopy and cluster analysis. For in vivo PS‐labeling 50 µg of the C1‐tetramer was i.v. injected. Additionally, EVs were stained in vitro for tetraspanins CD9 and CD81. Bar graphs show the number of in vivo stained PS+ (red, left graph), in vitro stained CD81+ and CD9+ (right graph) EV clusters mean ± SEM. Exemplary dSTORM micrographs are shown to the right. F) IFC analysis of splenocytes, shown is the gating strategy for splenic PS+CD19+ B cells and PS+CD11b+ monocytes/macrophages, exemplary images for EV/PS+ cells are shown to the right. G) Graph shows kinetics of in vivo labeled PS/EV+ splenocyte populations at 0, 15, 30, 60, 120, and 240 min as measured by IFC.

Based on our findings, we concluded that PS is widely exposed on nearly all plasma EVs in both mice and humans. We sought to utilize this characteristic to study the turnover of endogenous EVs in vivo using the C1‐tetramer. In previous studies, we demonstrated the effectiveness of both MFG‐E8 and the C1‐tetramer for in vivo labeling of cell‐bound EVs, and here we extend their application to analyze circulating unbound EVs simultaneously (**Figure** **4**A). First, we examined whether C1‐tetramer‐labeled EVs could be detected using imaging flow cytometry (IFC) following intravenous (i.v.) injection and subsequent EV isolation. EV‐controls, CFSE‐labeling, and gating are shown in Figure S9A–F (Supporting Information). To address the possibility that residual free C1‐tetramer might contribute to labeling, we evaluated the stability of the signal (Figure S9G–I, Supporting Information). Two EV samples were independently labeled with C1‐tetramer conjugated to distinct fluorophores and subsequently purified by SEC. Upon co‐incubation for 90 min, no co‐localization was observed, suggesting that free C1‐tetramer was effectively removed by SEC (Figure S9G,H, Supporting Information).

Next, we injected mice with 50 µg C1‐tetramer and isolated plasma EVs 15 min later. Approximately 75% of all EVs were labeled with the C1‐tetramer in vivo (Figure 4B), a frequency that was not elevated by increasing the dose of injected reagent (Figures S10 and S11D,E, Supporting Information).

In vivo–labeled PS⁺ EVs showed slightly lower frequencies than those from in vitro labeling (Figure 1, 2) or counterstaining (Figure 4B, “PS in vitro BV421”), likely due to reduced C1‐tetramer availability from blood flow dynamics or rapid clearance. No inhibition was observed between the C1‐tetramers at the concentrations used in our experiments (Figure 4B). As shown in Figure S10D,E (Supporting Information), titration of the same reagent labeled with distinct fluorophores revealed competition only at substantially higher concentrations, suggesting that epitope availability was not limiting under our conditions. The majority of EVs were successfully labeled in vivo, confirming the suitability of C1‐tetramer injection for studying blood EV turnover.

We next assessed the half‐life of PS^+^ plasma EVs via IFC at various time points. In vivo‐labeled PS^+^ EVs were completely cleared from circulation within 2 h (Figure 4C, left), with a calculated half‐life of 29 min (Figure S9E, Supporting Information). Importantly, in vivo PS labeling did not alter the overall population of PS⁺ plasma EVs, as confirmed by in vitro counterstaining for PS and CD9 (Figure 4C, right), which demonstrated a stable EV composition over time. Similarly, nanoparticle tracking analysis (NTA) at all time points revealed no changes in EV abundance (Figure 4D). We further validated in vivo labeling at 15 and 120 min using super‐resolution microscopy of PS‐captured EVs (Figure 4E; Figure S9F, Supporting Information). At 15 min, most EV clusters retained the in vivo PS label, whereas by 120 min, it was no longer detectable. Notably, CD81^+^ and CD9^+^ EV cluster counts remained constant over time.

Finally, we compared the turnover of circulating plasma EVs with that of cell‐bound EVs isolated from the spleen (Figure 4F,G). After i.v. injection of the C1‐tetramer or streptavidin‐AF647 as a negative control (Figure S11B, Supporting Information), we isolated splenocytes for IFC analysis. PS^+^ cells were predominantly EV^+^ with fewer than 1% apoptotic cells, as determined by a convolutional autoencoder machine‐learning model (Figure S11A, Supporting Information).^[^ ^20^ ^]^ We also included ApoA1/ApoE in our analysis and could confirm that PS did not co‐localize with lipoprotein markers on EV+ cells (Figure S11C, Supporting Information).

Next, we focused on splenic B cells (CD19^+^) and monocytes/macrophages (CD11c^+^) due to their high PS/EV positivity.^[^ ^31^ ^]^ Interestingly, we found that the turnover of cell‐bound EVs in spleen cells was prolonged compared to circulating EVs. PS/EV^+^ splenocyte frequency remained virtually unchanged over a 4‐h period (Figure 4G).

## 3 Discussion

In this study, we found that PS externalization is a predominant feature across various small EV populations in murine and human blood, including TSPN^+^ EV subsets, which have been previously described as partially or fully lacking external PS exposure.^[^ ^8^, ^19^ ^]^ The analyzed EVs, with a mean plasma size of 150 nm, were significantly smaller than apoptotic or necrotic bodies (1–5 µm; ^[^ ^32^ ^]^). PS is also exposed on apoptotic cells and bodies. To minimize cross‐reactivity, we employed multiple strategies: in vivo labeling to avoid artifactual binding to dying cells during organ preparation; viability dyes and size gating to exclude apoptotic cells and larger vesicles; SEC to enrich for small EVs; and a deep learning‐driven image interpretation to distinguish apoptotic cells from live cells with surface‐bound EVs.^[^ ^20^, ^21^, ^22^ ^]^ Additionally, the EVs analyzed were small and tetraspanin‐positive, whereas apoptotic bodies are typically larger and show variable or no tetraspanin expression.^[^ ^33^ ^]^ While we cannot fully exclude contributions from apoptotic processes, our data strongly support that most PS+ signals derive from bona fide small EVs. Additionally, we demonstrated that the detected PS^+^ vesicles were not primarily derived from major lipoprotein or chylomicron contaminants. Hence, exposed PS is widely present on most circulating EVs in healthy murine and human plasma, though not necessarily all EVs under all conditions. Our study is limited to EVs derived from human and murine plasma, as well as cell‐associated EVs from mouse spleen. Since EVs from different tissues and sources can vary considerably in their PS content, further studies are needed to determine whether EVs in other body fluids are also predominantly PS‐positive. However, recent lipidomics analyses have shown that plasma EVs contain 170‐ to 570‐fold less PS compared to EVs from milk, urine, saliva, or semen.^[^ ^19^ ^]^ Given that plasma EVs in our study were still largely PS‐positive, it is tempting to speculate that PS is indeed broadly distributed across diverse EV populations.

PS‐positive EVs can interact with various cell types, including phagocytes that recognize PS and help regulate EV turnover.^[^ ^20^, ^21^, ^22^ ^]^ We have also shown that EVs can modulate T cell responses if they carry relevant antigens or co‐stimulatory signals.^[^ ^22^ ^]^ Beyond these functions, PS itself may act as a driver of thromboinflammation by activating coagulation and complement cascades ^[^ ^34^ ^]^ (reviewed in ^[^ ^35^ ^]^). If PS+ EVs bind to viable immune cells, this may lead to cell damage and negative immunomodulation. Given that most circulating EVs are PS‐positive, it is conceivable that not only cell‐associated but also free PS+ EVs contribute to thromboinflammatory responses. This supports the potential of PS‐blocking agents‐ such as ours ‐as therapeutic tools to counteract such effects.

A marker that is reliably found on nearly all EV populations is crucial for standardized EV quantification, facilitates the monitoring of EV levels for diagnostic purposes, enhances the characterization of therapeutic EVs, enables the measurement of EV turnover and biodistribution, and deepens our understanding of EV biology.

However, comprehensive EV characterization remains essential. Further research is needed to elucidate PS externalization mechanisms in EV subtypes beyond apoptotic bodies and platelet‐derived EVs. Reduced flippase activity, possibly due to limited ATP production, may contribute to PS exposure in most EVs. Additionally, localized PS exposure has been linked to microvesicle formation via membrane curvature changes and increased TMEM16F activity.^[^ ^7^, ^8^, ^36^, ^37^ ^]^

The question of PS exposure on EVs has been a point of contention in the field, with studies reporting PS as characteristic of only select EV subpopulations,^[^ ^2^, ^38^, ^39^ ^]^ while others suggest that PS exposure is more widespread.^[^ ^19^, ^40^ ^]^ Here, we demonstrate that variability in PS‐binding protein affinity likely contributes to these discrepancies. Distinct affinities of Annexin V and MFG‐E8 for PS have been observed in previous studies, primarily in the context of platelets,^[^ ^41^, ^42^ ^]^; our study extends this comparison to liposomes, EVs, and cells. With EV flow cytometry, where low epitope density on EVs can complicate analysis, highly sensitive reagents are essential for accurate results.^[^ ^43^ ^]^

We observed that Annexin V exhibited low to negligible binding affinity for particles with low PS content, such as submicron‐sized liposomes (1–5% PS) and blood EVs, which have been described to contain PS at low frequencies.^[^ ^19^ ^]^ However, Annexin V effectively bound to PS on necrotic cells and large liposomes (1000 nm) with high PS content (50%), indicating that its affinity may depend on both PS density and particle size or membrane curvature. Our findings are consistent with previous reports indicating that Annexin V's anticoagulant function is reduced with PS levels below 15%.^[^ ^26^ ^]^ In contrast, MFG‐E8 demonstrated higher PS affinity, especially on EVs, while the C1‐tetramer showed the highest sensitivity across EVs and cells. Given the variability in PS frequencies across EV sources, further analysis on EVs with high PS levels (e.g., from breast milk or saliva) could help clarify binding characteristics. Additionally, our findings indicate that binding affinities for MFG‐E8 and C1‐tetramer are influenced by both particle size and PS density, with dissociation constants ranging from 4.5 to 96 nm, consistent with previously published data for full‐length MFG‐E8.^[^ ^44^, ^45^, ^46^ ^]^ A full understanding of factors influencing MFG‐E8 and C1‐tetramer binding affinities remains beyond this study, but careful titration of PS‐binding proteins is recommended for EV analysis. Our findings may thus guide future studies aiming to more precisely (re)evaluate altered PS levels on EVs, particularly in pathological contexts such as tumor‐derived EVs.

Leveraging the prevalence of PS on plasma EVs, we used MFG‐E8‐based reagents to study in vivo EV turnover. While prior studies have investigated EV clearance by reintroducing exogenously labeled EVs^[^ ^47^ ^]^ or using genetic markers to trace EV subpopulations,^[^ ^48^, ^49^ ^]^ our study offers a novel approach to track endogenous EV turnover. Exogenous EVs are cleared rapidly from circulation (95% within 5 min ^[^ ^50^, ^51^, ^52^ ^]^), whereas our data suggest a slower clearance rate for endogenous EVs (t_1/2_ = 29 min). This discrepancy might stem from differences in PS frequency or from PS‐independent clearance mechanisms. To our knowledge, this study is the first to employ broad in vivo labeling, enabling insights into the turnover of circulating and cell‐bound endogenous EVs in physiological and pathological conditions. Future research could elucidate whether reductions in serum EVs observed during acute infections, such as LCMV, are linked to altered turnover rates.^[^ ^22^ ^]^

Although we cannot entirely exclude the possibility that C1‐tetramer labeling affects EV clearance, we observed no reduction in PS exposure on labeled EVs, evidenced by consistent in vivo and in vitro PS co‐staining. This finding is relevant given PS's known role in EV uptake via multiple receptors,^[^ ^33^, ^53^, ^54^ ^]^ including Tim4, RAGE, Bai‐1, and stabilin‐2, all of which are expressed on macrophages essential for PS^+^ EV clearance.^[^ ^40^, ^50^ ^]^ Consistent with these observations, we found that PS^+^ EVs predominantly interact with CD11b^+^ monocytes/macrophages and CD19^+^ B cells in the spleen.^[^ ^55^ ^]^ Furthermore, our results showed that after clearance from circulation, endogenously labeled PS^+^ EVs persist on spleen cell surfaces and are likely internalized by macrophages. Splenic EV retention clearly outlasts their circulation time, likely due to interactions with PS receptors on target cells. However, currently, we cannot discriminate whether cell‐associated EVs are internalized or remain surface‐associated. Moreover, if these EVs persist or are released again, if they are transferred to other cells or are cleared, if their mRNA content or other biomolecules contribute to functional changes in target cells, or if most EVs have no biological relevance at all, is currently unknown and the topic of further studies in many laboratories. Studying EV kinetics in other tissues, particularly the liver, an organ known for macrophage‐mediated EV clearance, could provide further insights.^[^ ^40^ ^]^

Our study provides unprecedented detail showing that most in vivo EVs are PS+, enabling their targeted tracking for turnover analysis. This novel reagent opens new avenues for studying EV biogenesis, function, behavior, and the monitoring of therapeutically applied EVs.

## 4 Experimental Section

### Mice

All mice were housed and bred under specific pathogen‐free conditions at the Core Facility Animal Models of the Biomedical Center of the Ludwig‐Maximilians‐University, Munich. All protocols were approved by the Government of Oberbayern. Age‐matched female mice, 8–12 weeks old, were used. C57BL/6NRj mice were purchased from Janvier (strain C57BL/6). Mice received 50 µg of C1‐tetramer for the purpose of endogenous EV labeling.

### Antibodies/Reagents

Table S3 (Supporting Information).

### MFG‐E8 Based PS Staining Reagents

The C1‐tetramer was produced by mixing biotinylated mC1 (patent publication No. US 2024/0125806 A1^[^ ^56^ ^]^) with SA‐AF647 (BioLegend), SA‐BV421 (BioLegend), SA‐AF488 (Biolegend), or SA‐CF568 (Biotium) in a 5:1 molar ratio. The tetramer is available at Biolegend (Apotracker TM Tetra, Cat‐# 427405) and, when used, the manufacturer's protocol was followed. After tetramerization, the samples were centrifuged for 30 min at 20 000 g 4 °C to remove aggregates. Recombinant MFG‐E8‐eGFP was produced as previously described.^[^ ^20^ ^]^

### Blood and Cell‐Culture‐Derived EV Isolation

Mouse blood was collected via heart puncture, and coagulation was prevented by the addition of heparin (Ratiopharm). Plasma was separated by centrifugation at 1500 g for 10 min at 4 °C. Plasma was diluted to 800 µL in DPBS containing 1x protease inhibitor (Roche) and subsequently centrifuged two more times (2500 g, 10’, at 4 °C and 10 000 g, 10’, at RT) before application to qEV 35‐nm columns (Izon Science). Flow‐through was collected in 500 µL fractions. EV‐containing fractions were determined by NTA, BCA and Western blotting, pooled, and concentrated to 300 µL. Human plasma was collected from 5 healthy individuals after overnight fasting (3 males, 2 females, age 21–60) into S‐Monovette sodium heparin tubes and processed as described above for murine samples. The study protocol (Project‐Nr. 18‐0415) was approved by the Institutional Review Board of the Medical Faculty of LMU Munich.

For cell culture‐derived EVs, serum‐free adapted HEK293 cells were cultured in EX‐CELL 293 Medium (Sigma) containing 1% P/S, 20 mm HEPES and 200 µm L‐Glutamine at 37 °C. After 3 days, supernatant was collected and centrifuged sequentially at 2000 g at 4 °C and 10 000 g at RT for 10 min. Afterward, medium was concentrated 50‐fold and 1 mL of sample, containing 1X protease inhibitor cocktail (Roche), was added onto qEV 35‐nm columns (Izon Science). Flow‐through was collected in 500 µL fractions, and EV‐containing fractions were determined by NTA and BCA.

### BCA, SDS‐PAGE and Western Blotting

Protein contents were measured using a BCA protein assay kit (Thermo Scientific, Rockford, IL, USA) according to manufacturer's instructions, using BSA as a standard.

For Western blotting, the 12 EV fractions were pooled in pairs and concentrated to 200 µL. Each sample (20 µL) was denatured at 95 °C for 5 min in the presence of 1x loading buffer (50 mm Tris‐HCL pH 6.8, 100 mm DTT, 2% SDS, 0.1% Bromophenol blue, 10% glycerol). Samples were loaded onto 12% SDS‐PAGE for protein separation. Proteins were transferred onto nitrocellulose blotting membrane (Amersham, Cytiva) in a Mini Trans‐blot cell system (Bio‐Rad). After transfer, membranes were blocked with 1x Casein blocking buffer (Sigma–Aldrich) for 90 min at RT. Primary antibodies were diluted in 1x Casein blocking buffer and incubated overnight at 4 °C. After washing with TBS‐T, secondary antibodies were diluted in 1x Casein blocking buffer were added for 90 min at RT. Following washing with TBS‐T, Western Lightning Plus‐ECL (PerkinElmer) reagents were used for the detection of protein bands in an iBright 1500 imager (ThermoFisher).

### Superresolution Microscopy dSTORM

For direct stochastic optical reconstitution microscopy (dSTORM) analysis, 15 µL of EVs at 1‐5 x 10^10^ particles mL^−1^ were stained overnight at 4 °C with different combinations of antibodies (Table S3, Supporting Information) or the C1‐tetramer‐AF647 in the presence of 0.5% bovine serum albumin See Figure 2B, and Fc receptor blocking diluted in filtered DPBS. Purified antibodies were labeled in‐house with dSTORM compatible dyes using Mix‐n‐Stain CF488 and CF568 kits according to the manufacturer's instructions. EVs stained with isotype control antibodies and SA‐AF647, and antibodies diluted in DPBS without EVs were used as negative controls. Then, flow chambers were assembled consisting of SA‐coated glass slides (Ibidi) and 1.5H cover slides. Flow chambers were either coated with 0.3 mg mL^−1^ with commercially available C1‐tetramer (Apo‐Monomer, Biolegend #427405) for PS‐based capture or with CD81‐biotin (Biolegend #104903) at a final concentration of 0.25 mg mL^−1^, both for 15 min at RT. After coating, flow chambers were washed twice with 100 µL filtered DPBS before proceeding to EV capture for 45 min at RT. Washing was repeated as previously mentioned, and afterward, EVs were fixed with 4% paraformaldehyde for 10 min at RT, followed by another washing step. Freshly prepared BCubed STORM‐imaging buffer (ONI, Oxford Nanoimaging) was added before image acquisition on a temperature‐controlled Nanoimager S Mark II microscope from ONI. Images were taken in dSTORM mode, acquired sequentially using the total reflection fluorescence (TIRF) illumination (calculated evanescent field penetration depth was > 200 nm). Before imaging, channel mapping was calibrated using 0.1 µm TetraSpeck beads (T7279, Thermo Fisher Scientific). Superresolution images were filtered using the NimOS software (v.1.18.3, ONI) and data have been further processed with the Collaborative Discovery (CODI) online analysis platform from ONI. Post‐processing data analysis included correction for drift, temporal grouping and filtering to remove suboptimal localization and improve overall localization precision. For cluster analysis, HDBSCAN was used and at least 15 localizations were required to constitute a cluster.

### Imaging Flow Cytometry for EV Analysis

The imaging flow‐cytometer (IFC) ImageStream MKII (Cytek) requires small volumes of EV sample (25–200 µL). It was opted to use a staining volume of 60 µL and a final volume of 120 µL, adjusting buffer/reagent/antibody concentrations appropriately, since no washing steps could be performed following the labeling. The primary purpose of dilution was to balance between timely acquisition and potential complications associated with too high concentration ‐ for instance “swarm formation.”

All EV or liposome preparations analyzed by IFC were stained either with CFSE (carboxyfluorescein succiminidyl ester, eBiosciences) or CellTrace violet (Thermo Fisher), which were commonly used for labeling of membranous vesicles such as EVs. For platelet‐poor plasma (PPP) preparations, plasma samples were centrifuged twice (2500 x g, 10’ and 10 000 x g, 10’ at RT) and diluted 1:10 at this step and directly used for staining. Liposomes with defined size and DOPS:DOPC content were purchased from Encapsula NanoSciences. Prior to staining, EV or liposome concentration was determined using NTA analysis and adjusted to 1–5 × 10^10^ particles mL^−1^. All staining reagents were centrifuged at 18 000 g for 30 min at 4 °C. 1 µL of CFSE or CTV was added to 40 µL of sample material to reach a final concentration of 10 µm. After addition of the membrane dyes, samples were incubated for 1 h at RT protected from light. Antibodies or staining reagents were added to the samples (for concentrations see Tables S1 and S2, Supporting Information) in a total volume of 60 µL (to reach 60 µL filtered PBS was added if necessary). For staining, 6 µL of Annexin binding buffer (Annexin V 10x binding buffer, BD Pharmingen) were added to the samples while the total volume of 60 µL remained the same. Subsequently, samples were incubated for 1 h at RT, protected from light. Afterward, samples were split in half and 2.5% (v/v) NP‐40 was added to the detergent controls, whereas the same volume of PBS was added to the samples. Prior to IFC measurements, samples were diluted to 120 µL with filtered PBS or 120 µL filtered PBS containing 1x Annexin V binding buffer. Aggregate controls contained the same final concentration of antibodies, staining reagents or membrane dyes diluted in filtered PBS. Antibody specificity was tested for all antibodies using appropriate isotype controls or SA‐fluor for the C1‐tetramer. As negative controls, fluorescence minus one, unstained EVs, reagent/buffer, and detergent controls were used. For all panels used here, serial dilutions were performed to exclude bias stemming from “swarm detection”. According to the MISEV guidelines^[^ ^24^ ^]^ comprehensive list of all staining reagents, concentrations, and methodological procedures can be found in the (Tables S1 and S2, Supporting Information).

Before single vesicle analysis, the ability of the ImageStream MKII was tested to detect single‐size populations of fluorescent sub‐micron beads by measuring two commercially available mixtures of FITC‐fluorescent polystyrene (PS) beads of known sizes (Megamix‐Plus FSC–900, 500, 300, and 100 nm, and Megamix‐Plus SSC–500, 240, 200, and 160 nm). Next, both bead sets in a 1:1 ratio (‘Gigamix’) were mixed and performed acquisition and tested the ability of the ImageStream MKII to discern all seven fluorescent bead populations, as well as the 1 µm‐sized Speed Beads (SB), via the FITC (Ch02) and side scatter (SSC‐Ch06) intensities. For subsequent EV analysis, CFSE or CTV membrane labeling was used, and gated on low scatter, to exclude the instrument‐specific speed beads, CFSE/CTV^+^ events. Acquisition of EVs was performed with fluidics set at low speed, sensitivity set to high, magnification at 60×, core size 7 µm, and the “Remove Beads” option unchecked before every acquisition. The ImageStream MKII was equipped with the following lasers run at maximal power to ensure maximal sensitivity: 405 nm (120 mW), 488 nm (200 mW), 561 nm (200 mW), and 642 nm (150 mW). Upon each startup, the instrument calibration tool ASSIST was performed to optimize performance and consistency. Two channels (Ch01 and Ch09) were set to bright‐field (BF), permitting spatial coordination between cameras.

### Preparation of Single Cell Suspensions for Flow Cytometry

Single‐cell suspensions of the spleen were prepared by meshing the organs through a nylon mesh, followed by erythrocyte lysis using an ammonium‐chloride‐potassium buffer. 5 × 10^6^ cells were blocked with Fc block (10 min on ice) and stained for imaging flow cytometry with appropriate antibody mixes (25 min on ice), after the staining procedure cells were washed twice with DPBS and finally resuspended in 50 µL for IFC or 500 µL for analysis on the BD FACSCanto II (BD Biosciences). When staining with Annexin V, cells were stained, washed, and resuspended in 1x Annexin V buffer (BD Pharmingen) diluted with PBS. For IFC acquisition, cells were analyzed at low speed and 60× magnification on an ImageStream MKII. PS^+^ cells were identified using FMO controls, and for IFC analysis apoptotic and EV^+^ cells were digitally sorted using a machine‐learning based convolutional autoencoder (CAE) as previously described.^[^ ^20^ ^]^

### Nanoparticle Tracking Analysis

Particle number and size distribution of EVs were determined by NTA using the ZetaView PMX110 instrument (ParticleMetrix). Eleven positions were measured with three reading cycles with temperature control at 21 °C enabled. Preacquisition parameters were sensitivity = 75, shutter speed = 50, frame rate = 30 fps, trace length = 15. Postacquisition parameters were minimum brightness = 20, pixels size = 5 to 1000.

### Multiplex Bead‐Based EV Surface Protein Profiling

Human or murine purified EV fractions were analyzed using MACSplex exosome kit (MACSplex Exosome kit human/mouse, Miltenyi) for multiplex analysis by flow cytometry. All samples were adjusted to 1–2 × 10^10^ particles per assay and incubated with MACSplex exosome capture beads for 18 h on an orbital shaker at 450 rpm at room temperature. Beads were washed according to the manufacturer's protocol with either PBS/BSA 1%(w/v) or for Annexin V staining, PBS/BSA 1%(w/v)/ 1× Annexin V binding buffer. For staining of the captured EVs, different concentrations of Annexin V‐AF647 (Biolegend) or the C1‐tetramer‐AF647 were used. As controls, served beads without EVs, beads with EVs but no PS‐labeling (PS FMO), or beads with EVs stained with SA‐AF647. All staining reagents were incubated with the samples for 1 h on an orbital shaker at 450 rpm and RT. Afterward, samples were washed twice and finally resuspended in 300 µL PBS/BSA 1% (w/v) (with or without Annexin V binding buffer). All samples were acquired on a BD FACSCanto II (BD Biosciences). Geometric mean fluorescent intensities (MFIs) for all capture bead subset were background‐corrected by subtracting respective MFI values from matched non‐EV‐containing buffer controls. MFI values below those obtained for EVs stained with SA‐AF67 of each respective sample were reported as ‘not detected’ for C1‐tetramer staining. For Annexin V, an Annexin V FMO control was used for the same purpose.

### Statistical Analysis

For statistical analysis, the PRISM software (GraphPad Software) was used. All data were tested for normal distribution using the Shapiro‐Wilk normality test. Significance was analyzed using the Student's t test or one‐way ANOVA test unless stated otherwise, with ns *P*> 0.05, ^*^ *P* < 0.05, ^**^ *P* < 0.01, and ^***^ *P* < 0.001. To correct for multiple comparison, Holm Sidak method was used. Graphs show average ± SEM. Dissociation constants (kD) were calculated by fitting non‐linear regression curves with the Hill slope equation in PRISM. The goodness of the fit was evaluated by R^2^ values, which ranged from 0.82 to 0.99. The half‐life of in vivo labeled plasma EVs was determined by fitting a non‐linear one phase decay curve in PRISM. The goodness of the fit was evaluated by R^2^ (0.91).

## Conflict of Interest

T.B. and J.K. declare competing interests due to an exclusive licensing agreement with BioLegend, Inc.

## Author Contributions

J. K. and T.B. contributed equally to this work. L.F. performed experiments and wrote the 1st draft of the manuscript; M.F., A.K., and W.H. provided reagents and performed experiments; C.R., G.K., and M.S. performed and supervised experiments; A.K. provided material and expertise; J.K. and T.B. designed the study, wrote the paper.

## Supporting information



Supporting Information

Supporting Information

## Data Availability

The data that support the findings of this study are available from the corresponding author upon reasonable request.
