# Supplementary material for: Assessing Extracellular Vesicle Turnover In Vivo Using Highly Sensitive Phosphatidylserine‐Binding Reagents
Source: Adv Sci (Weinh). 2025 Aug 16;12(40):e07624. doi: 10.1002/advs.202507624 (PMC12561326; doi:10.1002/advs.202507624)
Supplement: Supplementary file 1 — Supporting Information [file ADVS-12-e07624-s002.docx]

**SUPPLEMENTARY FIGURES**

**Assessing Extracellular Vesicle Turnover In vivo Using Highly Sensitive Phosphatidylserine-Binding Reagents**

Lavinia Flaskamp^1^, Monica Prechtl^1^, Annkathrin Scheck^1^, Wenbo Hu^1^, Christine Ried^1^, Georg Kislinger^2^, Mikael Simons^2^, Anne Krug^1^, Jan Kranich^1#^, Thomas Brocker^1#^

^1^Institute for Immunology, Faculty of Medicine, BMC, LMU Munich, Großhaderner Strasse 9, 82152 Planegg

^2^﻿German Center for Neurodegenerative Diseases (DZNE), Munich, Germany

**Supplementary Figures**

|  |
| --- |

**Suppl. Fig. S1: (A)** Megamix-Plus (BioCytex) SSC and FSC beads with distinct submicron sizes analyzed by imaging flow cytometry (IFC), all bead populations, ranging from 100 nm to 900 nm could be resolved based on scatter and fluorescent emission in Ch02 on the ImageStream^TM^. SB, speed beads. **(B-E)**, Imagestream^TM^ analysis of liposomes (Encapsula), shown are exemplary graphs for aggregate controls at 5 nM and 125 nM of all staining reagents in **(B)** and **(C)**, respectively, unstained liposomes are depicted in **(D)** and PS FMO liposomes stained only with CTV are shown in **(E)**. **(F)**, Exemplary twofold serial dilution for all liposome populations stained with CTV and either Annexin V (FITC), MFG-E8-eGFP or the MFG-E8 C1-Tetramer (AF488) at 5 nM, measured by IFC. Linear regression analysis (grey dashed lines) was performed to check for swarm detection, goodness of fit is indicated by R-square values. **(G)** Elution profile of cell culture EVs from serum-free adapted HEK293 cells, briefly, EVs were isolated via SEC and the collected fractions were analyzed by nanoparticle tracking analysis (NTA) for particle concentration/mL (lines) and by bicinchoninic acid assay (BCA) to determine protein content in each fraction (bars). **(H)** Size distribution analysis of pooled fractions (F2-6). **(I)** Gating on SSC_low_/CFSE+ events for unstained HEK293 EVs and aggregate controls for all staining reagents. **(J)** HEK293 EVs were isolated (n=4) and analyzed by IFC for PS positivity of CD9+ (orange box) and CD9- EVs (blue box). **(K)** Matched isotype control for CD9 and SA-AF647 as a negative control for the C1-Tetramer. **(L)** Twofold serial dilution of HEK293 EVs stained with CFSE, C1-Tetramer and CD9, measured by IFC. Linear regression analysis (red dashed line) was performed to check for swarm detection, goodness of fit is indicated by R-square values.

|  |
| --- |
| **Suppl. Fig. S2:**  **(A)** Elution profile of murine plasma EVs unlabelled or labelled with the C1-Tetrarmer prior to size exclusion chromatography (SEC), briefly, EVs were isolated via SEC and the collected fractions were analysed by nanoparticle tracking analysis (NTA) for particle concentration/mL (lines) and by bicinchoninic acid assay (BCA) to determine the protein content in each fraction (bars). **(B)** Western blot of SEC fractions for negative control serum albumin and the EV marker CD9 as a positive control. **(C)** Size distribution analysis of pooled fractions (F1-5) for unlabelled vs. labelled plasma EVs. **(D)** Co-staining of membrane dyes, CTV and CFSE, used for IFC analysis of liposomes and EVs. Exemplary graphs shown for controls of plasma EV IFC analysis (main Fig. 1G) including unstained EVs, detergent controls and aggregates controls for murine plasma EVs labelled with 110 nM of **(E)** C1-Tetramer (AF647) **(F)** Annexin V (AF647) or **(G)** MFG-E8-eGFP. **(H)**, Staurosporine-treated (1 µg/ml, 3 h) apoptotic murine (C56BL/6 mice) thymocytes were stained either with Annexin V or MFG-E8 C1-Tetramer or both reagents together in Ca^2+^-containing or Ca^2+^-free buffer as indicated and analyzed by flow cytometry. |

| **** |
| --- |
| **Suppl. Fig. S3:**  **(A-C)** Twofold serial dilution of plasma EVs (main Figure 1 G,H) stained with CFSE or CTV and different concentrations of **(A)** C1-Tetramer (AF647), **(B)** Annexin V (AF647) **(C)** MFG-E8-eGFP (n=3), measured by IFC. Linear regression analysis (red dashed line) was performed for each dilution series to check for swarm detection, goodness of fit is indicated by R-square values. |

|  |
| --- |
| **Suppl. Fig. S4:** **(A)** Gating strategy for splenocytes analysed by flow cytometry. Briefly, cells were heat-shocked for 3 min at 60°C to induce cell death and increase PS exposure. **(B)** PS-labelling of total, necrotic (LD+) or apoptotic/EV+ (LD-) splenocytes using distinct concentrations of Annexin V-FITC (black), C1-Tetramer-AF647 (red) or MFG-E8-eGFP (blue) (n=3). **(C)** Histograms for PS fluorescent intensities of Live-Dead (LD)+ versus - splenocytes stained with different PS-binding reagents. |

| 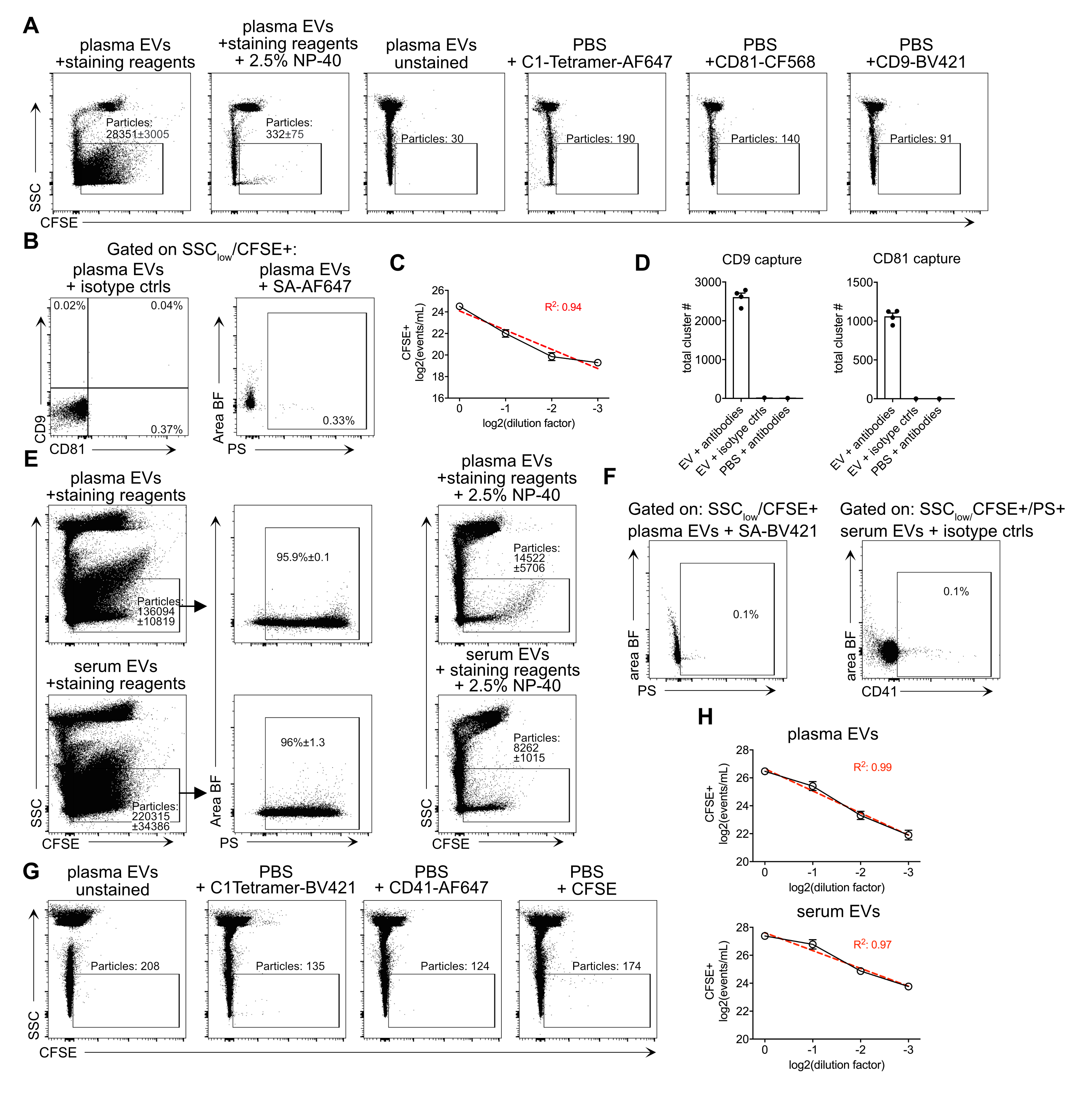 |
| --- |
| **Suppl. Fig. S5:** **(A)** Gating on SSC_low_/CFSE+ events for plasma EVs (main Fig. 2A) and detergent controls (n=3) as well as unstained EVs and aggregate controls for all staining reagents. **(B)** Matched isotype controls for CD9 and CD81 antibodies and SA-AF647 staining as a negative control for the C1-Tetramer-AF647 **(C)** Twofold serial dilution of plasma EVs stained with CFSE, C1-Tetramer, CD9 and CD81, measured by IFC. Linear regression analysis (red dashed line) was performed to check for swarm detection, goodness of fit is indicated by R-square values. **(D)** Antibody/C1-Tetramer only ('PBS+antibodies') and matched isotype controls ('EVs + isotype ctrls') for dSTORM analysis of murine EVs using different capturing reagents (main Fig. 2B), shown is the number of total clusters as determined by cluster analysis. **(E)** Gating on SSC_low_/CFSE+ and PS+ murine serum or plasma EVs and detergent controls (n=3) for main Fig. 2D. (**F)** Matched isotype control for CD41-AF647 and SA-BV421 staining as a negative control for the C1-Tetramer-BV421. (**G)** Gating on SSC_low_/CFSE+ events for unstained EVs and aggregate controls for all staining reagents used in main Figure 2D. **(H)** Twofold serial dilution of plasma EVs stained with CFSE, C1-Tetramer and CD41 measured by IFC. Linear regression analysis (red dashed line) was performed to check for swarm detection, goodness of fit is indicated by R-square values. |

| 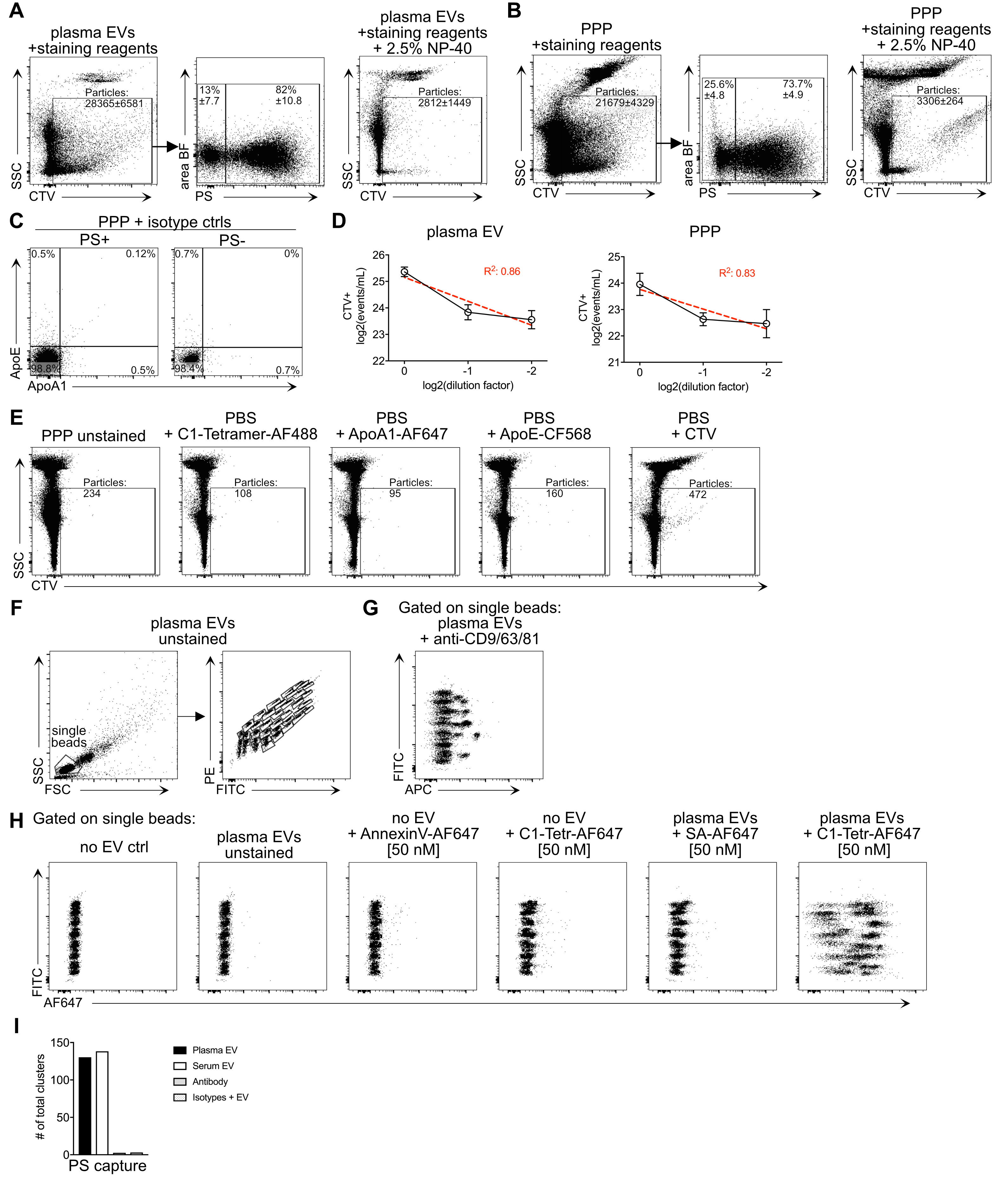 |
| --- |

**Suppl. Fig. S6:** **(A)** Gating on SSC_low_/CTV+ events and analysis of PS positivity for murine platelet poor plasma (PPP) or SEC-isolated plasma EVs (main Fig. 2E) and detergent controls (n=4) as well as unstained PPP and aggregate controls for all staining reagents in **(E)**. **(C)** Matched isotype controls for ApoA1 and ApoB **(D)** Twofold serial dilution of murine platelet poor plasma (PPP) or SEC-isolated plasma EVs stained with CTV, C1-Tetramer and ApoA1/B, measured by IFC. Linear regression analysis (red dashed line) was performed to check for swarm detection, goodness of fit is indicated by R-square values. **(F)** Exemplary gating strategy for bead-assisted flow cytometry as shown in Figure 2F. Single beads are first gated by SSC/FSC-A and individual beads containing antibodies directed against typical EV surface markers then separated by their endogenous PE/FITC-signal, enabling discrimination of 35 bead populations. **(G)** Detection antibody mix (Miltenyi) was used as an internal assay control.**(H)** AF647 fluorescent intensity of background controls ('no EV ctrl', 'no EV + Annexin V', 'no EV + C1-Tetr.', 'plasma EVs unstained' and 'plasma EVs + SA-AF647') versus plasma EVs detected by C1-Tetramer-AF647 labelling. **(I)** Antibody only and matched isotype controls for dSTORM analysis of murine PS+ EVs (main Fig. 2F), shown is the number of total clusters as determined by cluster analysis.

|  |
| --- |
| **Suppl. Fig. S7:**  **(A)** Elution profile of human plasma EVs, briefly, EVs were isolated via SEC and the collected fractions were analyzed by nanoparticle tracking analysis (NTA) for particle concentration/mL (lines) and by bicinchoninic acid assay (BCA) to determine protein content in each fraction (bars). **(B)** Western blot of SEC fractions for negative control serum albumin and the EV marker CD9 as a positive control. **(C)** Size distribution analysis of pooled fractions (F1-5). **(D)** Gating on SSC_low_/CFSE+ events for human plasma EVs (main Fig. 3A) and detergent controls (n=5) as well as unstained EVs and aggregate controls for all staining reagents. **(E)** Matched isotype control for CD9 and SA-AF647 as a negative control for the C1-Tetramer **(F)** Twofold serial dilution of human plasma EVs stained with CFSE, C1-Tetramer and CD9, measured by IFC. Linear regression analysis (red dashed line) was performed to check for swarm detection, goodness of fit is indicated by R-square values.**(G)** Antibody only and matched isotype control for dSTORM analysis of human EVs (main Fig. 3B), shown is the number of total clusters as determined by cluster analysis. **(H)** Gating on SSC_low_/CTV+ events for human PPP or SEC-isolated plasma EVs (main Fig. 3C) and detergent controls (n=4) as well as unstained PPP and aggregate controls for all staining reagents. **(I)** Analysis of PS positivity among detected events in human plasma EVs versus PPP. **(J)** Matched isotype control for ApoB-CF568 and ApoE-CF488. **(K)** Twofold serial dilution of human plasma EVs or PPP stained with CTV, C1-Tetramer and ApoB/ApoE, measured by IFC. Linear regression analysis (red dashed line) was performed to check for swarm detection, goodness of fit is indicated by R-square values. |

|  |
| --- |
| **Suppl. Fig. S8: (A)** Exemplary gating strategy for bead-assisted flow cytometry as shown in main Fig. 3C. Single beads are first gated by SSC/FSC-A and individual beads containing antibodies directed against typical EV surface markers then separated by their endogenous PE/FITC-signal, enabling discrimination of 39 bead populations. **(B)** Detection antibody mix (Miltenyi) was used as an internal assay control. **(C)** AF647 fluorescent intensity of background controls ('no EV ctrl', 'plasma EVs unstained' and 'plasma EVs + SA-AF647') versus plasma EVs detected by C1-Tetramer-AF647 labelling. |

| 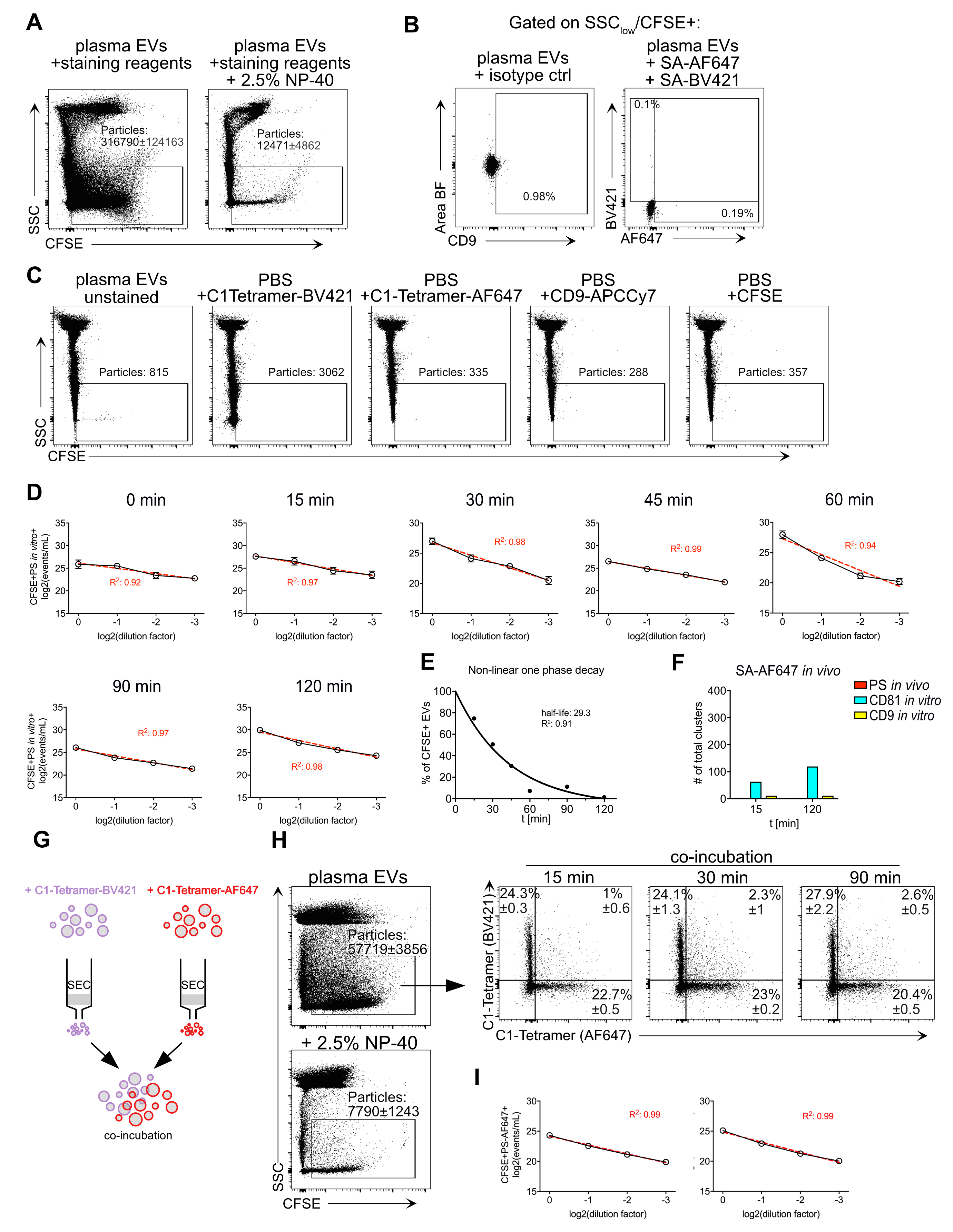 |
| --- |
| **Suppl. Fig. S9:**  **(A)** Gating on SSC_low_/CFSE+ events for *in vivo* labelled plasma EVs (t=15 min, n=5)(main Fig. 4B) **(B)**Matched isotype control for CD9 and SA-BV421 staining as a negative control for the C1-Tetramer *in vitro* staining. **(C)** Exemplary graphs after 15 min of C1-Tetramer i.v. injection, showing detergent controls (n=5) as well as unstained EVs and aggregate controls for all staining reagents. **(D)** Twofold serial dilution of *in vivo* PS-labelled plasma EVs stained with CFSE, C1-Tetramer *in vitro/in vivo* and CD9 measured by IFC. Linear regression analysis (red dashed line) was performed to check for swarm detection, goodness of fit is indicated by R-square values. **(E)** Half-live of in vivo labelled plasma EVs (main Fig. 4C) was determined by fitting a non-linear one phase decay curve. **(F)** EV clusters at two timepoints (15 and 120 min) after i.v. injection of SA-AF647. Bar graphs show number of *in vivo* stained SA+ (red), *in vitro* stained CD81+ (cyan) and CD9+ (yellow) EV clusters. **(G,H)** To assess stability of the C1-Tetramer EV labelling, separate plasma EV samples were stained with C1-Tetramer in BV421 or AF647. Afterwards free dye was removed by SEC and the distinctly labelled EVs were pooled and incubated at 36°C for 15, 30 or 90 min (n=3). To check for dye transfer, EVs were analyzed by IFC. **(I)** Twofold serial dilutions and linear regression analysis (red dashed line) was performed for the C1-Tetramer labelled EVs to control for swarm detection. Goodness of the fit is indicated as R-square values. |

| 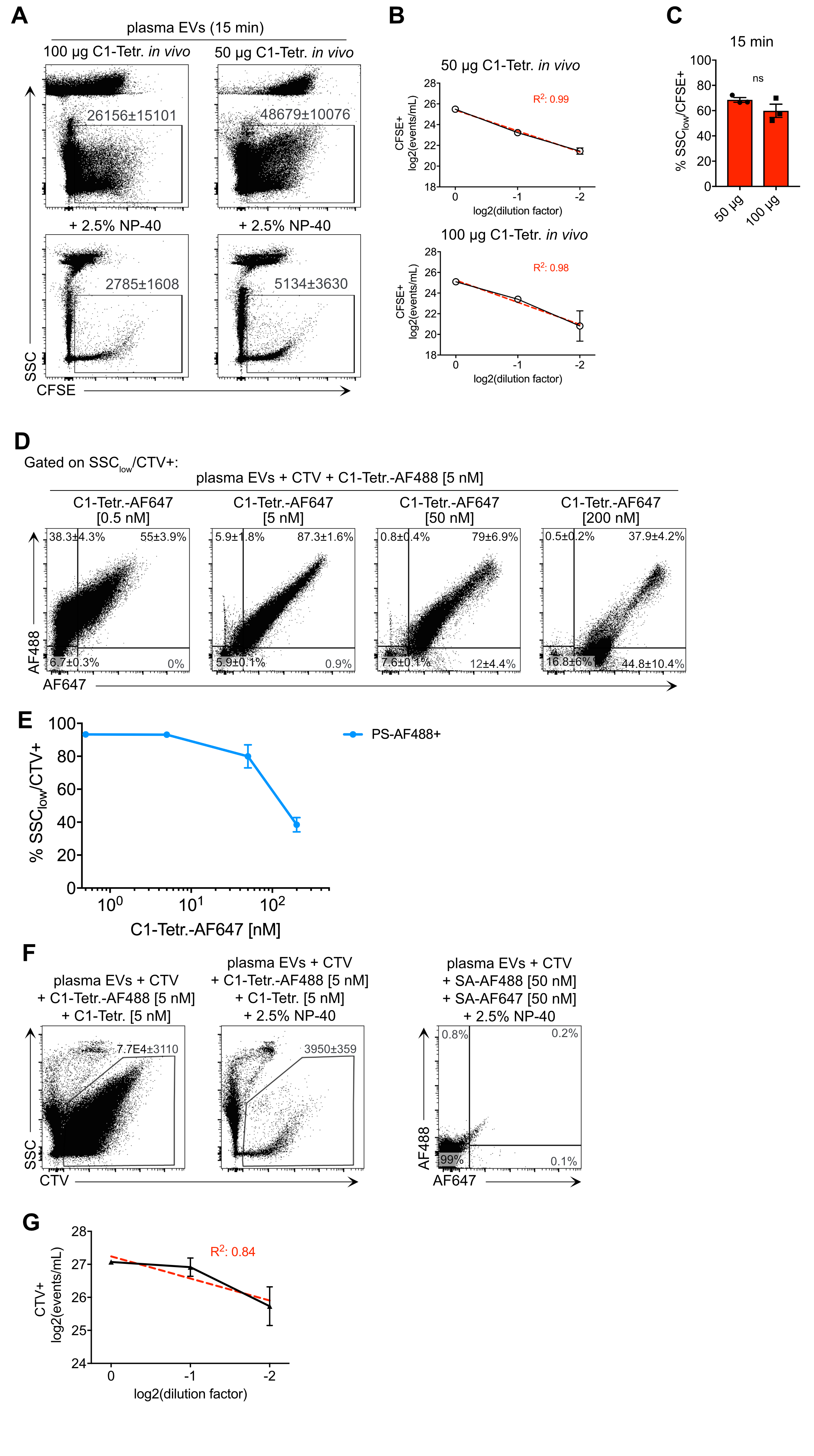 |
| --- |
| **Suppl. Fig. S10:** *In vivo* labelling efficiency by C1-Tetramer (AF647) was assessed by injecting 50 versus 100 µg and plasma EVs were analyzed by IFC (n=3). Gating strategy and detergent controls are shown in (**A**) and twofold serial dilutions with linear regression analysis (red dashed line) was performed to check for swarm detection. Goodness of the fit is indicated as R-square values (**B**). (**C**) Comparison of *in vivo* labelling of plasma EVs after 15 mins using two different C1-Tetramer concentrations. Normality of the data was tested for by Shapiro-Wilk normality test and an unpaired t test was done to test for significance (p > 0.05, ns). (**D**)To check for PS blocking by addition of the C1-Tetramer, plasma EVs were stained with CTV and a fixed concentration (5 nM) of C1-Tetramer (AF488) for 1h. Afterwards, C1-Tetramer labelled with another fluorphore (AF647) was added at increasing concentrations **(D)**. **(E**) Competition of first C1-Tetramer (AF488) is shown. (**F**) Gating on SSC_low_/CTV+ events for plasma EVs and detergent controls (n=3) as well as EVs stained with SA-AF488 and SA-AF647 as controls. **(G)** Exemplary twofold serial dilution of plasma EVs stained with CTV, C1-Tetramer (AF488 [5 nM] and AF647 [50 nM]) measured by IFC. Linear regression analysis (red dashed line) was performed to check for swarm detection, goodness of fit is indicated by R-square values. |

| 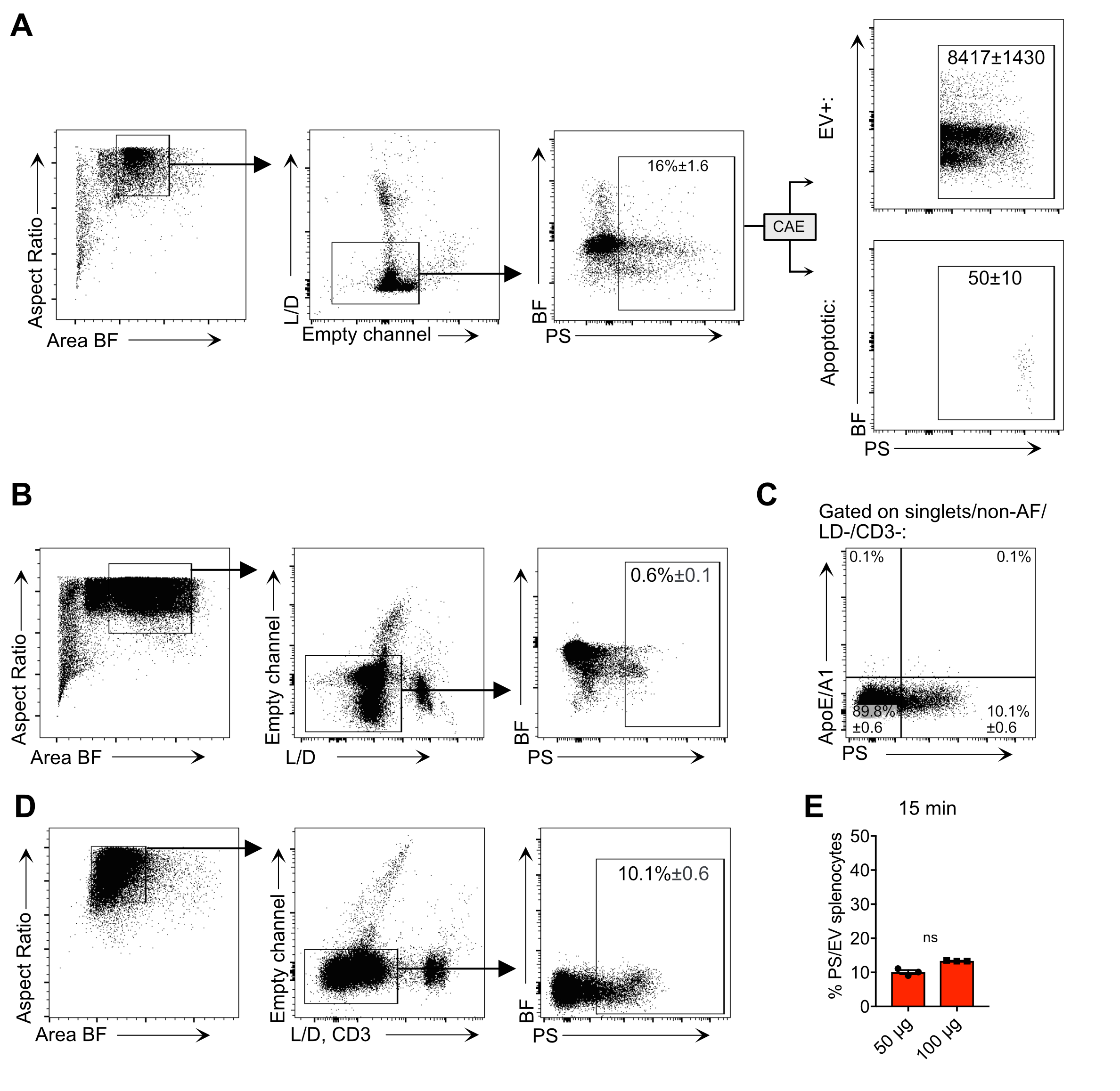 |
| --- |
| **Suppl. Fig. S11:**  **(A)** To discriminate dying and EV+ cells, murine splenocytes were analyzed using IDEAS, a machine-learning based convolutional autoencoder (CAE) and FlowJo, as described previously in Chlis and Kranich et al., 2020; Rausch et al., 2021; Rausch et al., 2023. Exemplary analysis shown for t = 30 min. **(B)** Control mice were injected with 50 μg SA-AF647 (n=3) and splenocytes were analyzed by IFC after 15 min, to check for unspecific labelling. (**C**) To test for the presence of PS+ lipoprotein particles attached to cells rather than EVs, splenocytes were stained for ApoE/ApoA1 and potential co-localization with PS was examined (n=3), 15 min post-injection of 50 µg C1-Tetramer (AF647).**(D)**,**(E)** *In vivo* labelling efficiency by C1-Tetramer (AF647) was assessed by injecting 50 versus 100 µg and splenocytes were analyzed by IFC (n=3). Exemplary gating strategy for splenocytes **(D)**. Comparison of EV+ cells after 15 mins using two different C1-Tetramer concentrations. Normality of the data was tested for by Shapiro-Wilk normality test and a Mann-Whitney test was done to test for significance (p > 0.05, ns) **(E)**. |

**References**

Kranich, J., Chlis, N. K., Rausch, L., Latha, A., Schifferer, M., Kurz, T., Foltyn‐Arfa Kia, A., Simons, M., Theis, F. J., & Brocker, T. (2020). *In vivo* identification of apoptotic and extracellular vesicle‐bound live cells using image‐based deep learning. *Journal of Extracellular Vesicles*, *9*(1), 1792683. <https://doi.org/10.1080/20013078.2020.1792683>

Rausch, L., Flaskamp, L., Ashokkumar, A., Trefzer, A., Ried, C., Buchholz, V. R., Obst, R., Straub, T., Brocker, T., & Kranich, J. (2023). Phosphatidylserine-positive extracellular vesicles boost effector CD8 ^+^ T cell responses during viral infection. *Proceedings of the National Academy of Sciences*, *120*(16), e2210047120. <https://doi.org/10.1073/pnas.2210047120>

Rausch, L., Lutz, K., Schifferer, M., Winheim, E., Gruber, R., Oesterhaus, E. F., Rinke, L., Hellmuth, J. C., Scherer, C., Muenchhoff, M., Mandel, C., Bergwelt‐Baildon, M., Simons, M., Straub, T., Krug, A. B., Kranich, J., & Brocker, T. (2021). Binding of phosphatidylserine‐positive microparticles by PBMCs classifies disease severity in COVID‐19 patients. *Journal of Extracellular Vesicles*, *10*(14), e12173. <https://doi.org/10.1002/jev2.12173>
